# A protocol for precise comparisons of small vessel disease lesions between ex vivo magnetic resonance imaging and histopathology

**DOI:** 10.1177/1747493018799962

**Published:** 2018-09-10

**Authors:** Catherine A Humphreys, Maurits A Jansen, Susana Muñoz Maniega, Víctor González-Castro, Cyril Pernet, Ian J Deary, Rustam Al-Shahi Salman, Joanna M Wardlaw, Colin Smith

**Affiliations:** 1 Academic Neuropathology, University of Edinburgh, Edinburgh, UK; 2 Centre for Clinical Brain Sciences, University of Edinburgh, Edinburgh, UK; 3 Neuroimaging Sciences, University of Edinburgh, Edinburgh, UK; 4 Department of Electrical, Systems and Automatic Engineering, Universidad de León, León, Spain; 5 Department of Psychology, University of Edinburgh, Edinburgh, UK; 6 UK Dementia Research Institute at the University of Edinburgh, Edinburgh, UK

**Keywords:** Brain, magnetic resonance imaging, histology, small vessel disease, cerebrovascular disorders

## Abstract

**Rationale:**

Neuroimaging and clinical studies have defined human sporadic cerebral small vessel disease but the pathophysiology remains relatively poorly understood. To develop effective therapies and preventative strategies, we must better understand the heterogeneity and development of small vessel disease at a cellular level.

**Hypothesis:**

Small vessel disease lesions as seen on neuroimaging have specific neuropathological correlates.

**Methods and design:**

Standard histological samples are taken from strategic areas of the brain typically affected by small vessel disease, in cases with a range of disease from mild to severe and controls. Tissue is formalin fixed, scanned using 7-tesla magnetic resonance imaging and processed for histology. Histological slides are digitalized then registered with the corresponding magnetic resonance image. Small vessel disease burden is assessed and lesions are precisely identified on the ex vivo imaging and microscopy independently then compared. The tissue can be interrogated using multiple magnetic resonance sequences and histological methods targeting the gliovascular unit.

**Study outcomes:**

The primary outcome is identifying and defining the cellular characteristics of small vessel disease lesions compared to imaging. Secondary outcomes are related to obtaining information about abnormalities of protein expression in the gliovascular unit, defining groups of small vessel disease severity in our cohorts for future analysis and developing a reliable, reproducible protocol for accurate radiological–histological lesion comparison, which can be applied to other neurological diseases in the future.

**Discussion:**

Comprehensive, precise pathological–radiological–clinical correlations in small vessel disease will provide greater insight into associations and pathophysiology underlying magnetic resonance imaging findings in normal- and abnormal-appearing tissue, ex vivo and in vivo.

## Introduction

Sporadic human cerebral small vessel disease (SVD) is common, causing 25% of all ischemic strokes,^1^ 85% of all intracerebral haemorrhage^2^ and vascular dementia. SVD is seen in at least 56.5% of Alzheimer's disease^3^ and synergistically worsens symptoms.^4^ Although the neuroradiological characterization of SVD lesions has been standardized,^5^ similar approaches are needed in pathological assessment of SVD, and the pathological basis of radiological lesions remains poorly understood.

Pathological studies are not dynamic. They are limited to tissue taken at the time of death. Much of the pathological literature has focused on the lacune, which is relatively easily identified at autopsy, but is descriptive.^6^ Attempting to understand the pathophysiology is difficult;^7^ pathological studies, especially with imaging correlations, are few,^8,9^ with little information on histopathological changes in and around SVD lesions seen on imaging and whether lesions vary by brain region.^10^ Systematic reviews of studies comparing post-mortem imaging and histology appearances^8,11^ reveal just three studies correlating 79 microbleeds identified on magnetic resonance imaging (MRI) with histopathology in SVD in 15 cases,^12–14^ while one other paper described three cases with at least three microbleeds.^15^ Five papers studied lacunes: 2 with 59 lacunes in 18 cases,^16,17^ one with four cases and at least four lacunes,^18^ one with two cases and unknown numbers of lacunes^19^ and one study describing lacune appearances without any details of numbers of cases or lesions.^20^ There are greater numbers of studies looking at white matter hyperintensities (WMH), but their pathophysiology has not yet been fully eluciated.^21,22^

All previous post-mortem studies aiming to characterize SVD lesion appearances image whole brains, whole hemispheres or brain slices (manuscript in preparation). Some studies have attempted to provide more detailed localization by scanning both whole brains and individual macroscopic coronal brain slices.^10,23^ This is of value when assessing overall disease burden, but lesion locations are typically approximated using gross landmarks^24^ or tissue is sampled broadly from areas identified on imaging, precluding accurate comparison of individual lesions.^25^ As such these studies cannot confidently correlate histology with imaging. This may, at least partly, explain the limited understanding of SVD pathogenesis, the lesion heterogeneity at a cellular level, and the modest associations described so far between imaging and clinical features.^8^

We developed a protocol to precisely compare the histological and radiological features of SVD in human post-mortem brain tissue, to test the hypothesis that different SVD lesions identified and defined on MRI represent specific histological, lesions. We aim to use this information to define the histological lesions of SVD and compare it to clinical and in vivo radiological data. In the future we will further explore the pathophysiological mechanisms using targeted genetic studies, with the intention of ultimately being able to refine SVD animal models and identify therapeutic targets and preventative strategies.

## Methods

### Design

This is a prospective observational study using tissue from three cohorts representing severe SVD (Lothian study of INtraCerebral Haemorrhage, Pathology, Imaging and Neurological Outcome, LINCHPIN^2^), normal aging (Lothian Birth Cohort 1936, LBC1936;^26^ range from normal to severe SVD, including lesions in evolution) and controls (no or only mild SVD) within the Medical Research Council (MRC) Edinburgh Brain Bank.

### Study population

#### Inclusion criteria

- LINCHPIN^27^ and LBC1936^28^ defined by specific cohort criteria.
- Controls: sudden, unexpected, non-suspicious deaths with no known neurological disease in life.

#### Exclusion criteria

- Time from death to autopsy >5 days.
- Next of kin decline authorization.

#### Post-mortem neuropathological examination

**Figure 1.**
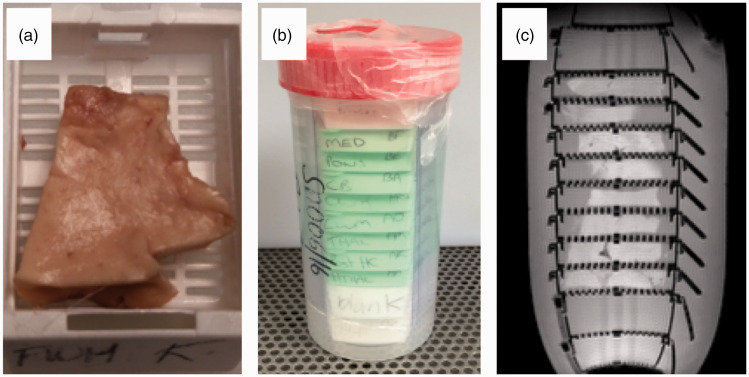
Small tissue samples are placed in plastic cassettes for standard histological processing (a). Eight cassettes are stacked in a plastic container and fixed (b) before scanning in a small bore 7T MRI scanner (c) (gradient echo scout). Tissue is only placed in the cassettes where the gradient coil is mostly linear, empty cassettes at each end and folded plastic along the length of the stack prevent movement within the container.

A standard neuropathological post-mortem examination is carried out by at least one neuropathologist according to Brain Bank protocols (unpublished), extended for this study to include areas typically affected by SVD on MRI. The brain is weighed, the cerebellum and brainstem are removed and 1 cm thick coronal sections are made. The cerebellum is sliced sagittally and the brainstem axially. Samples approximately 2 × 2×1 cm are taken from defined neuroanatomical areas (Table 1). Tissue from the left cerebral hemisphere only is used, as SVD is usually considered symmetrical.^5^ The right hemisphere will also be studied in 10% of cases, randomly chosen, to ensure this symmetry is true in our population. Each sample is bisected in the same plane as it was cut; one piece is placed in a plastic cassette and fixed in 10% unbuffered formalin in a plastic tube for 24–72 hours before MR scanning and histology (Figure 1). The complementary sample is frozen in nitrogen vapor for long-term storage at −150℃ to support future research applications.

**Table 1.** Areas affected by maximal radiological SVD in life selected for MR-histology comparison

*Areas studied*
• Anterior frontal parasagittal cortex (BA9)
• Broca's area (BA44/45)
• Temporal tip (BA38)
• Frontal, temporal, parietal, and occipital white matter
• Caudate nucleus
• Basal ganglia
• Hippocampus
• Hypothalamus
• Thalamus
• Cerebellum
• Pons
• Medulla

BA: Brodmann area.

#### Post-mortem magnetic resonance imaging

Through pilot work, we developed the most practical approach to satisfy both our requirements to make precise histopathological comparisons and to obtain excellent quality images. To minimize air bubble artefact, the lid of the container is secured under formalin cover and left to stand for 5 minutes, it is tapped to release trapped air and then the lid is re-secured under formalin. The cassettes are held by pieces of plastic at the sides with empty cassettes at each end to prevent movement.

The tissue is scanned overnight in a 7T small-bore rodent MRI scanner (Agilent Technologies, Yarnton, UK) equipped with a 400 mT/m gradient insert with sequences similar to those used in vivo to detect evidence of SVD (Table 2). The long acquisition time (135 minutes) increases sensitivity to small subtle features. For all scans (except the scout), the field of view is 60 × 60 mm, the slice thickness is 1 mm with no gap orientated axially, resolution 0.23 × 0.23 × 1.0 mm, 30 slices are obtained across the cassettes containing the tissue. For the scout scan, the field of view is 120 × 120 mm, slice thickness 2 mm with a 2 mm gap in three planes. Shimming is done on the entire plastic container using an automated routine for global shimming followed by manual adjustment of the shim coils. This optimizes the homogeneity of the magnetic field to avoid artefact on images. Quality of shim was checked by measuring the 50% linewidth of the H_2_O peak in an unlocalized ^1^H spectrum. Typical values were 50–60 Hz.

**Table 2.** MR imaging sequences and their parameters

Sequence	Scout	T2	T2*	T1	FLAIR
Type	GE	FSE	GE	FSE	FSE
TE (ms)	3.9	53	22	9.2	40
TR (ms)	23.3	2500	801	700	8000
TI (ms)					1816
Flip angle (°)	20	90/180	20	90/180	90/180
Echo spacing (ms)		13.25			20
Echo train length		4			2
Band width (Hz)	52k	78k	50k	100k	50k
Matrix	256 × 256	256 × 256	256 × 256	256 × 256	256 × 256
NEX	2	8	6	4	4
Acquisition time (minutes)	0.2	21.3	20.5	12	68.4

FLAIR: fluid attenuated inversion recovery; GE: gradient echo; FSE: fast spin echo; TE: echo time; TR: repetition time; TI: inversion time; NEX: number of signal averages.

We are not aware of any established grading systems for imaging tissue blocks and grading the SVD burden therein. We have therefore adapted existing protocols for assessing SVD in whole brain MRI^5^ which were developed, validated and applied in our studies of SVD including LBC1936 and LINCHPIN,^29–31^ and have been tested in these studies over the past two decades. Our existing protocols for assessing SVD on MRI are now included in international standard definitions.^5^ Disease burden assessment on MR and histology is performed independently and blinded to clinical and all other data.

#### Histology

After scanning, the formalin-fixed tissue is paraffin-embedded in its entirety and sections are stained based on previously published data from histological and imaging literature to assess the gliovascular unit and general neuroinflammatory and glial responses (Table 3). SVD burden,^32^ Alzheimer's disease pathology,^33,34^ a-synuclein^35^ and amyloid angiopathy^36^ are graded using published, validated systems. Slides are digitally scanned using the Xeiss Axioscan system.

**Table 3.** Special stains and immunohistochemical stains used for assessment of SVD, vascular and neurodegenerative pathology

Stains	Concentration	Antigen retrieval	Manufacturer	Feature assessed
*Cut at 4 μm*				
BA4	1:100	Formic acid	Dako, Glostrup, Denmark, M0872	Amyloid plaques, cerebral amyloid angiopathy
Tau	1:2500	None	Thermo Fisher Scientific, Illinois, US, MN1020	Neurofibrillary tangles
α-Synuclein	1:200	Pressure cooker and citric acid	Thermo Fisher Scientific, Illinois, US, AHB0261	Lewy bodies
Myelin basic protein	1:500	Formic acid	Abcam, Ab77895	Myelin
Neurofilament heavy	1:500	Citric and formic acid	Abcam, Ab8135	Axons
CD163	1:1000	Pressure cooker and citric acid	Abcam, Ab87099	Macrophages
Claudin-5	1:250	Pressure cooker and citric acid	Abcam, Ab15106	Endothelial tight junctions
Collagen-1	1:1000	Pressure cooker and citric acid	Abcam, Ab90395	Vascular media
Platelet-derived growth factor receptor β	1:100	Pressure cooker and citric acid	Abcam, Ab32570	Pericytes
Smooth muscle actin	1:1000	Pressure cooker and citric acid	Dako, Glostrup, Denmark, M0851	Vascular smooth muscle
CD68	1:100	Pressure cooker and citric acid	Dako, Glostrup, Denmark, M0876	Activated microglia
Glial fibrillary acidic protein	1:800	None	Dako, Glostrup, Denmark, Z0334	Astrocytes
*Cut at 6 μm*				
H&E				General structure
Masson trichrome				Connective tissue
Luxol fast blue				Myelin
Perl's				Iron

H&E: hematoxylin and eosin.

#### Image registration and analysis

The MR slice that best matches the histology section is selected visually, and the corresponding slice for each MR sequence is transformed into NIfTI format.

**Figure 2.**
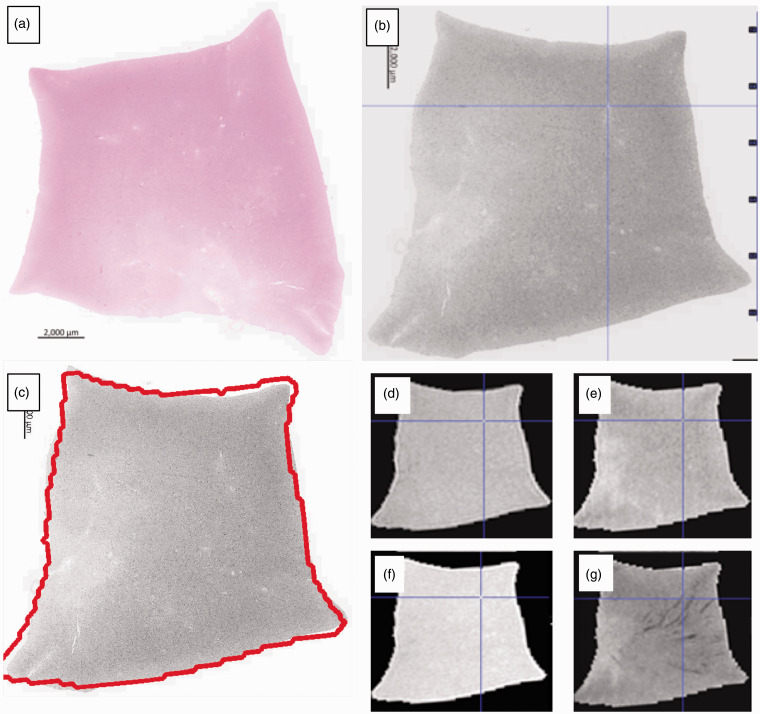
A sample of frontal white matter, where the lack of anatomical landmarks can make comparison difficult. The digitalized histology slide (a, H&E) is reoriented and registered (b) with the FLAIR (d), T2 (e), T1 (f) and T2* (g) MR sequences. An initial “quick check” image is produced with the MR outline overlaid on the registered histology (c) to confirm successful registration. Features of interest are selected on any image using the blue crosshairs and the precise corresponding feature on the other images is identified automatically. In this example, a prominent vessel on the MR sequences (d–g) and histology (b) has been identified.

The size of the histology image varies but is of the order of 10–20 thousand pixels in each dimension. To facilitate registration, they are rescaled by a factor of 1/20 using a Mitchell–Netravali kernel^37^ and converted to grey levels. To ensure successful registration, the histology images need to be reoriented to match roughly the position of MR images, which might involve flipping the image around the x or y axis. The histology samples are cut to be approximately rectangular in shape and kept in alignment with the rectangular cassettes. Due to the standardized nature of the histological processing methods, there are only a limited number of opportunities for it to be manipulated and rotated away from the position in which it was scanned. Therefore, there are only four possible orientations of the histology sample. The reorientation is done automatically by creating the four possible orientations of the histology image (original, flipped in x, flipped in y, flipped in x and y), registering each to the T1-weighted image (detailed below) and calculating a similarity score (normalized mutual information) between the histology and MR in each case. The highest similarity score corresponds to the best match between histology and the T1-weighted and therefore permits automatic selection of the correct histology orientation. An image with the outline of the MR overlaid on the histology is produced for each sample to enable a rapid visual confirmation of whether the registration has been successful (Figure 2). The automatic reorientation gives a satisfactory registration in >85% of the cases, in the cases where it fails, the histology is reoriented manually before registration, which then completes successfully.

**Figure 3.**
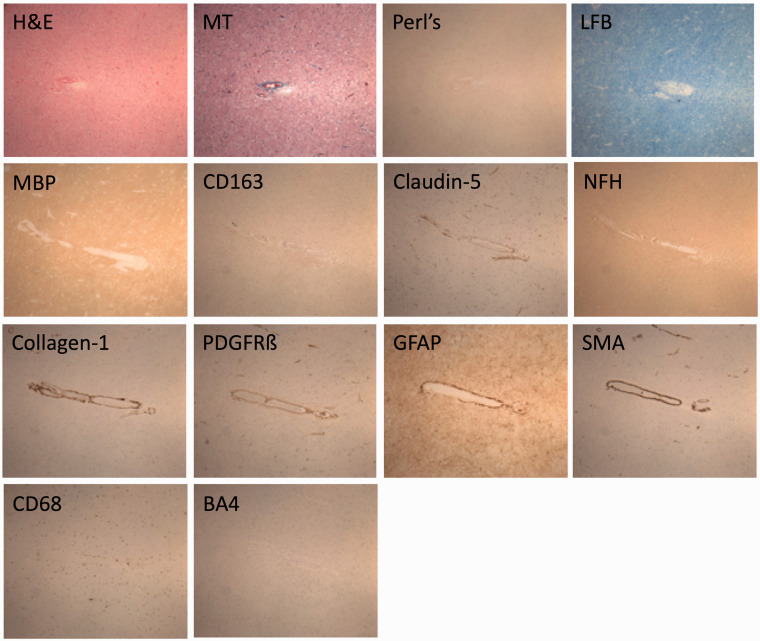
The white matter vessel identified in Figure 2 is stained (Table 3) to interrogate the gliovascular unit and blood–brain barrier (×40 magnification). As the course of the vessel is followed on consecutive histological sections the distribution and pattern of pathology can also be described.

Automatic registration of MR to each histology image is performed as follows. First, the T1-weighted image is used to create a binary mask of the tissue using k-means clustering^38^ with three clusters. The binary mask was created by setting the pixels in the cluster of the maximum center to 1 and the remaining pixels in the image to zero. This mask is applied to all MR images to exclude background signal and then cropped to the minimum field-of-view containing the tissue. Linear registration is then performed between the T1-weighted and histology images using NiftyReg over five levels of progressively finer resolution, with default settings.^39^ The transformation obtained is applied to the other MR modalities of the same slice, to obtain their registered versions (Figure 2). All registered MR images and the resampled histology are then saved in NIfTI format for visualization (MRIcro^40^). All processing is performed in R (v. 3.4) within RStudio (v. 1.0), using the packages “jpeg”, “divest”, “mmand”, “RNifti”, and “RNiftyReg”.^41–45^ The registered images are visualized and SVD lesions identified and described during grading are selected in one modality and automatically identified in the other, which allows accurate for characterization and comparison across all MR sequences and histological stains (Figures 2 and 3).

Scanning is done overnight on two consecutive nights, the registration is automated and lesion comparison takes several hours, depending on the number of lesions identified. The whole process from autopsy to lesions being compared takes about one week. Both MR and histology quality are excellent; histology, in particular is unaffected by the scanning process (examples in Figures 2 and 3).

In establishing this protocol, we have carefully considered how to minimize bias. All consecutive cases donated to the brain bank that meet the specific cohort and brain bank criteria are coded and processed using standardized procedures. Cases are batched until there are between 5 and 10, which are then rated at the same time. MR and histology ratings are carried out independently, blinded to cohort, clinical data and all other diagnoses and are based on validated guidelines as far as possible. A proportion of ratings will be carried out by a second experienced reviewer, and after a period of time repeated by the original reviewer, to allow inter- and intra-rater scores, respectively, to be calculated.

### Study outcomes

#### Primary outcomes

- Identify and define the pathological lesions of SVD on tissue sections, in relation to the lesions seen on neuroimaging.

#### Secondary outcomes

- Further understanding of gliovascular unit abnormalities from protein expression studies using additional stains and immunohistochemistry.
- Group cases by SVD severity as graded on MRI and histology for future assessment of protein and gene expression.
- Develop a reproducible protocol that can be adapted to study clinical–radiological–pathological correlations in other neurological diseases.

#### Estimates of likely numbers of cases

We estimate that about 18 brains will be donated and can be scanned per annum for all three cohorts in total, based on previous years. We began scanning in 2015 and have carried out this protocol in 53 cases so far; 21 severe SVD, 9 normal aging and 23 controls. Of these, 25 have also had in vivo MRI or CT brain imaging (17 severe SVD, 8 normal aging). In comparison with existing literature, this sample is one of the largest to date, and the only one to precisely and systematically study the areas most affected by SVD on in vivo imaging. It will allow for much greater depth of analysis regarding, for example, spatial variation in lesions, perilesional changes and severity of lesions. Numbers will increase as we continue to scan cases and include new cohorts; a population with symptomatic lacunar stroke^46^ and a subgroup of the Scottish Dementia Research Interest Register^47^ with Alzheimer's disease have recently been added.

#### Statistical analyses

Specific statistical analyses will depend on the distribution of the data; they will include descriptive statistics for lesion prevalence and appearances, and regression analyses for differences between groups. We will choose a number of relevant features to study from the rich clinical, imaging and histological data available.

For example, LBC1936 subjects have serial research-standard MRI in life with detailed assessment of SVD features including quantitative structural and functional analysis, medical histories and cognitive information from age 11 to the 8th decade.^26,29^ LINCHPIN includes current cognition, medical history and recent in vivo imaging. This will enable us to study associations where information is currently missing; such as the burden of SVD lesions visible in life and the prevalence of matrix protein abnormalities; the relationship between perivascular space visibility on MRI and numbers of active microglia; and confirm if the health of the myelin and axons in apparently normal tissue varies with the severity of WMH as is suggested by in vivo MRI.^48^ Control cases have medical and social history available. However, by definition they have had no neurological disease in life, have no cognitive impairment and therefore do not have in vivo imaging. We will be able to compare these data to the extent and severity of SVD assessed on the ex vivo imaging and histology. The extent of the analyses will be decided based on the final number of cases obtained. Inter-rater reliability will be assessed using weighted Kappa statistics.

After MRI and histology images are fully assessed, cases will be stratified into groups with and without cognitive impairment, by medical history and SVD burden, from none to severe. We will use the complementary frozen tissue to make targeted studies of gene expression differences between the groups. In particular, the short tissue fixation period retains RNA and DNA integrity supporting novel techniques such as BaseScope, a modified, quantifiable in situ hybridization technique identifying transcripts at a single cell level.^49^

#### Data monitoring body, study organization, funding and ethics

CAH is funded by an Alzheimer's Society Clinical Training Fellowship, and pilot work was supported by a Princess Margaret Research Development Fellowship through the Stroke Association. Post-mortem MR scanning was supported by a pilot grant from the MRC Centre for Cognitive Ageing and Cognitive Epidemiology and the Scottish Imaging Network (MRC Grant No. MR/K026992/1), A Platform for Scientific Excellence (SINAPSE) Initiative. JMW is supported by the EU H2020 PHC-03-15 project no 666881, SVDs@Target and Fondation Leducq project 16 CVD 05. IJD is supported by the Centre for Cognitive Ageing and Cognitive Epidemiology, which is funded by the MRC (Grant No. MR/K026992/1) and Biotechnology and Biological Research Council. The Lothian Birth Cohort 1936 is funded principally by Age UK (Disconnected Mind programme), and also the MRC (MR/M01311/1).

Informed consent is obtained from all participants in the severe SVD (ethical approval from Scotland A Research Ethics Committee ref. 10/MRE00/23) and normal aging cohorts in life (Multi-Centre Research Ethics Committee for Scotland MREC/01/0/56, Lothian Research Ethics Committee LREC/2003/2/29 and Scotland A Research Ethics Committee 07/MRE00/58, REC REF AM17, 07/MRE00/58/AM14).

Post-mortem authorization is also obtained from next of kin for all cases. The MRC Edinburgh Brain Bank has full ethical approval and consent for the use of tissue in research (East of Scotland Research Ethics Service, ref 16/ES/0084) and works within the framework of the Human Tissue (Scotland) Act 2006. It has a local management group and a steering committee, both of which include lay representation.

## Discussion

In our current study, two cohorts, LBC1936 and LINCHPIN, have several important complementary characteristics relevant to SVD; and other cohorts are now being added. The MRC Edinburgh Brain Bank is a responsive tissue resource, ensuring tissue is fit for end-user needs, developed around existing clinical cohorts, with high brain donation rates within the clinical cohorts.^27,50^ We employ targeted sampling with all residual tissue being returned to the body, as donation of small tissue samples is less distressing to relatives than whole organs.^51^ The Edinburgh Brain Bank is part of the UK Brain Bank Network, a coordinated group who have developed minimum diagnostic datasets, tissue handling protocols, and ethical and governance standards across multiple UK brain banks.^52^ Data linkage is key to maximizing this resource, and targeted post-mortem MRI adds to the data generated from each individual case.

We encountered several issues while developing this protocol. Air bubbles within the tissue and plastic cassettes cause MR artefact and may be mistaken for, or mask, microbleeds on T2* sequences making it important to minimize their presence, as described. Previous studies did not precisely match lesions on MR with histological images due to differing slice thicknesses and resolutions. We obtain serial 1 mm MR slices through each tissue block, although this is of a different order of thickness to the histological sections (mm versus microns). To make the most accurate comparison, the MR images are compared to corresponding histological images and the most complementary are registered. Formalin fixation of human brain tissue alters the MR properties of the tissue with time as the formalin diffuses into it.^53^ The use of small samples minimizes the difference in fixation between the deep and superficial tissue. T2 values of human brain tissue change with duration of fixation but plateau between 10 hours and five weeks.^53^ We have standardized tissue fixation to minimize fixation-induced variability between MR images. At 7T, tissue is vulnerable to overheating, which impairs tissue quality. Brain absorbs more radiofrequency than other tissues and our samples are in a relatively small amount of formalin without in vivo mechanisms, such as vasodilation, to dissipate heat. The scanner bore is cooled to 22.6℃ but this may not be sufficient. Our scanning protocols have been designed to maximize anatomic resolution and increase the signal to noise ratio while avoiding overheating. Temperature monitoring during scanning showed a maximum 1.8℃ rise within the plastic tube, which would not affect tissue quality and heat artefact has not been observed on histological examination.

As with many post-mortem studies, the number of cases available for study are relatively small. However, our study is one of the largest detailed post-mortem studies of SVD, and benefits from detailed data linkage and well-characterized cohorts. In addition, we employ extensive sampling of each case, targeting areas particularly affected by SVD and retaining complimentary frozen tissue for further interrogation with genomic technologies. The specific areas studied may be refined in future depending on the specific disease or characteristic under investigation. Pilot work within our lab has shown that formalin fixed paraffin-embedded tissue is not suitable for post-mortem imaging so retrospective imaging of cases with paraffin-embedded tissue is not possible. However, the histological protocol developed from the combined radiological–histological assessment could be applied retrospectively to appropriate tissue within the Brain Bank with relevant clinical data, thereby further extending the sample size for future related work using biochemical and genetic methods.

SVD lesions are well characterized on neuroimaging^5^ and described on neuropathology (although not consistently);^54^ however, attempts to correlate the appearances have found only modest associations.^8^ This may reflect variation in methodologies or true variation as suggested in rodent models where it appears SVD may result from multiple minor defects in different components of the neurovascular unit^55^ which we aim to study further with this protocol and future work. As a static assessment of a dynamic disease process, histopathological assessment may be limited; lesions at post-mortem in elderly patients are often end-stage. However, longitudinal assessments in life allow plotting of disease trajectories, and our initial work shows a gradation from subclinical, to lesions-in-evolution to severe lesions which will further inform interpretation of SVD pathophysiology. WMH in particular may be more dynamic than previously appreciated.^21^ Histopathological studies in humans have found protein expression abnormalities within the gliovascular unit in subjects with WMH^56^ and in vivo imaging suggests blood–brain barrier (BBB) dysfunction and leakiness.^30^ Alterations in the gliovascular unit are seen in an animal model prior to the onset of recordable hypertension.^57^

## Summary and conclusions

This protocol describes a new method to precisely assess and compare lesion appearances between brain MRI and histopathology to validate in vivo MRI findings in and around SVD lesions, aiming to increase our understanding of SVD pathophysiology and facilitate a meaningful clinical and radiological diagnosis. These methods and tissue may also be used in other conditions where the pathophysiology is poorly understood such as autism or the neurological effects of type 2 diabetes mellitus, and to validate clinical and radiological assessments and diagnoses.
